# Helical tomotherapy for chemo‐refractory multiple liver metastases

**DOI:** 10.1002/cam4.2651

**Published:** 2019-10-29

**Authors:** Taiki Takaoka, Yuta Shibamoto, Taro Murai, Masanori Kobayashi, Chikao Sugie, Yoshihiko Manabe, Takuhito Kondo, Dai Okazaki, Yuki Yamada, Akira Torii

**Affiliations:** ^1^ Department of Radiology Nagoya City University Graduate School of Medical Sciences Nagoya Japan; ^2^ Department of Immunotherapy Seren Clinic Nagoya Nagoya Japan

**Keywords:** chemo‐refractory, dendritic cell‐based vaccine therapy, helical tomotherapy, intensity‐modulated radiation therapy, multiple liver metastases

## Abstract

**Background:**

Despite advances in chemotherapy, curing multiple liver metastases is quite rare. Even when response is obtained, regrowth of the tumors is almost inevitable. We aimed to evaluate the efficacy and adverse events of helical tomotherapy for chemo‐refractory multiple liver metastases.

**Methods:**

Forty‐five patients with chemo‐refractory multiple (3‐10) liver metastases after standard systemic chemotherapy entered the single‐institutional prospective study. Liver metastases were the major disease; however, 31 also had uncontrolled primary lesions and/or other metastases. The prescribed dose was 55 Gy in 25 fractions. The median planning target volume (PTV) and normal liver volume (NLV) of first treatment were 128 cm^3^ and 1175 cm^3^, respectively. The median of V_15Gy_, V_30Gy_, and mean dose to NLV were 45%, 23%, and 19.4 Gy, respectively.

**Results:**

Forty‐two patients (93%) completed the planned treatment. Median survival time (MST) for all patients was 8 months, and the 1‐year survival rate was 29%. The median local control (LC) period was 5 months and the 6‐month control rate of irradiated tumors was 33%. A ≥30% decrease in tumor markers was observed in 31%. The most common grade 3 toxicity was lymphocytopenia (40%), followed by fatigue (6%). Radiation‐induced liver disease (RILD) was not observed. Pancreatic cancer as the primary tumor, distant metastases outside the liver, low pretreatment neutrophil‐to‐lymphocyte ratio (NLR), and low pretreatment monocyte‐to‐lymphocyte ratio (MLR) were associated with poorer prognoses.

**Conclusions:**

Helical tomotherapy for chemo‐refractory multiple liver metastases is a feasible and potentially effective treatment. Incorporating tomotherapy into the first‐line treatment in combination with systemic chemotherapy should be considered.

**Trial registration number:**

CROG 12005.

## Graphical abstract

Despite the remarkable advances of systemic chemotherapy for multiple liver metastases, the efficacy is still limited and cure of the multiple lesions is difficult. Helical tomotherapy for chemo‐refractory multiple liver metastases was suggested to be a feasible and potentially effective treatment with acceptable adverse events.

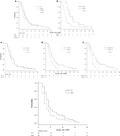

## 1 INTRODUCTION

The gold standard of treatment for multiple liver metastases is systemic chemotherapy, and radiation therapy (RT) has rarely been used. Although stereotactic body RT (SBRT) is efficient for hepatic oligometastases,^1^, ^2^ it is not indicated when uncontrolled primary tumor or other metastases exist. SBRT is often used for patients with a single liver metastasis without extrahepatic lesions and occasionally used for patients with two liver metastases in Japan; however, SBRT is rarely used for three or more liver metastases. Thus, systemic chemotherapy remains the main treatment for such patients. In recent years, systemic chemotherapy, including molecular targeted therapy, has remarkably advanced, and favorable responses of multiple metastases to chemotherapy are often observed. However, the efficacy is still limited and curing multiple lesions is quite rare; even when a partial or complete response is achieved, the regrowth of the tumors is almost inevitable. Furthermore, the long‐term adverse events are often intolerable, making the continuation of chemotherapy difficult. Because of these adverse effects, some patients refuse chemotherapy.

In these situations, physicians have to abandon chemotherapy, but there is no other effective treatment, and the expected survival time is usually several months or less. Attending physicians usually recommend hospice care; however, most patients and their families wish to receive further intensive treatment especially in Japan. For such patients, our group previously proposed a combination of dendritic cell (DC)‐based vaccine therapy and intensity‐modulated RT (IMRT).^3^ DC is a specialized family of professional antigen‐presenting cells that drive T‐lymphocyte‐mediated immune responses.^4^ In our previous study, we observed that IMRT had marked local effects even against chemo‐refractory cancers, and helical tomography could potentially treat multiple metastatic tumors.^3^

Based on these previous investigations, we started to use helical tomotherapy for patients with chemo‐refractory multiple liver metastases. Our aim was to delay tumor progression and prolong survival time. After evaluating toxicity of helical tomotherapy in several patients with multiple metastases or hepatocellular carcinomas with different fractionation schedules, we initiated this prospective study to evaluate the efficacy and toxicity of helical tomotherapy for multiple liver metastases. The combination with DC‐based immunotherapy was not mandatory, since the treatment is not covered by medical insurance. The purpose of this study was to evaluate treatment outcomes for chemo‐refractory multiple liver metastases.

## 2 MATERIALS AND METHODS

### 2.1 Study design and eligibility

This study was approved by the institutional review board at Nagoya City University Hospital (No. 1304) and was conducted in compliance with the guidelines of the Helsinki Declaration. The primary endpoint was overall survival (OS) and the secondary endpoints were local control (LC), progression‐free survival (PFS), safety, and toxicity. The inclusion criteria were as follows: (1) primary lesions diagnosed as malignant solid tumors; (2) age ≥20 years; (3) Eastern Cooperative Oncology Group (ECOG) performance status (PS) of 0‐2; (4) number of liver metastases 3‐10; (5) absence of malignant ascites; (6) history of systemic chemotherapy and chemo‐refractory status; (7) no indication of SBRT; (8) no previous irradiation to the liver; (9) normal liver volume (NLV) ≥ 700 cm^3^; (10) Child‐Pugh class‐A liver function; (11) no organ at risk (OAR) of exceeding dose constraints; (12) tumor volumes outside the liver less than 1/3 of the volume of liver metastases; and (13) written informed consent. The presence of an active primary lesion was allowed. Ambiguous, small (<1 cm) lesions were allowed to be unirradiated; the number of these unirradiated lesions was 1‐5 (median, 3) in 14 patients, but the volumes of the lesions were less than 5% of those of the total liver tumors. From our experiences, the maximum number of liver metastases was considered to be 10, provided that they were not too large. The exclusion criteria were as follows: (a) active infectious disease; (b) severe psychological disorder; (c) expected survival time <2 months estimated using the prognosis based on palliative care study predictor models.^5^ Combined use with DC‐based immunotherapy was allowed, since it did not seem to shorten or markedly elongate survival time in patients with highly advanced cancers.

Assuming a 6‐month OS rate of 50% for the treatment group compared with 20% for the best supportive care group,^6^ at least 43 patients were required based on a type‐1 error of 5%, a type‐2 error of 20%, and a drop‐out rate of 10%. Therefore, the sample size in this study was 45 patients.^7^ Since this was a single‐institution study and the treatment was unfamiliar to many surgeons and medical oncologists, we assumed 5 years would be necessary to accrue this number of patients, expecting an accrual of 8‐10 patients per year.

### 2.2 Patients

Between January 2013 and March 2018, 45 eligible patients with chemo‐refractory multiple liver metastases entered this study. All patients had become resistant to standard systemic chemotherapy regimens; 14 patients had only liver metastases, and 31 patients had active primary lesions and/or distant organ metastases outside the liver (lymph node, lung, bone, and/or peritoneum). The patient and tumor characteristics are shown in Table 1. The median interval of systemic chemotherapy before tomotherapy was 10 months (range, 5‐38) for all patients, 7 months (range, 5‐15) for patients with pancreatic cancer, and 12 months (range, 5‐38) for patients with the other primary cancers.

**Table 1:** Patient, tumor, and treatment characteristics (n = 45)

Age (y)	
Median (range)	64 (40‐93)
Gender	
Male/female	25/20
ECOG performance status	
0‐1/2	29/16
Primary tumor site	
Pancreas/Colon/Liver and bile duct	15/9/7
Stomach/Duodenum/Breast/Esophagus	3/3/2/2
Lung/Soft tissue/Uterus/Ovary	1/1/1/1
Number of liver metastases	
3/4/5‐10	5/2/38
Distant organ metastases outside the liver	
Absent/Present	14/31
Concurrent DC‐based vaccine therapy	25
Systemic chemotherapy before tomotherapy	
Pancreas (n = 15)	
GEM/S‐1/GEM + nab‐PTX/FOLFIRINOX	15/9/2/1
Others (n = 30)	
FOLFOX/FOLFIRI/GEM/S‐1	7/5/5/5
XELOX + Bev/FP/FEC/CDDP + VP‐16	3/2/2/1
TP/TC/Nexavar/CDDP + S‐1/DOX	1/1/1/1/1

Abbreviations and standard drug doses: DC, dendritic cell, ECOG, Eastern Cooperative Oncology Group; GEM, Gemcitabine (1000 mg/m^2^); S‐1, Tegafur, Gimeracil, and Oteracil (60‐100 mg/m^2^); nab‐PTX, nab‐Paclitaxel (125 mg/m^2^); FOLFIRINOX, Leucovorin + 5‐Fluorouracil + Irinotecan + Oxaliplatin (200, bolus 400/2400, 180, 85 mg/m^2^); FOLFOX, Leucovorin + 5‐Fluorouracil + Oxaliplatin (100, bolus 400/ 600, 85 mg/m^2^); FOLFIRI, Leucovorin + 5‐Fluorouracil + Irinotecan (200, bolus 400/ 2400, 150 mg/m^2^); XELOX + Bev, Capecitabine + Oxaliplatin + Bevacizumab (130, 2000 mg/m^2^, 7.5 mg/kg); FP, Fluorouracil + Cisplatin (700, 70 mg/m^2^); FEC, 5‐Fluorouracil + Epirubicin + Cyclophosphamide **(**500, 100, 500 mg/m^2^); CDDP + VP‐16; Cisplatin + Etoposide (80, 100 mg/m^2^); TP, Paclitaxel + Cisplatin (67.5, 50 mg/m^2^); TC, Paclitaxel + Carboplatin (175 mg/m^2^, Area Under the Curve 6); Nexavar (Sorafenib, 800 mg/d**);** CDDP + S‐1 (60, 80 mg/m^2^); DOX, Doxorubicin (20 mg/m^2^).

### 2.3 Radiotherapy protocol

The BodyFIX system (Medical Intelligence, Schwabmuenchen, Germany) was used for immobilization and minimizing the respiratory movements of targets. All patients were trained to breathe shallowly. Non‐contrast and contrast‐enhanced CT images were acquired using a 16‐row multi‐slice CT (Optima CT 580W; General Electric), with 2.5‐mm slice thicknesses. Contouring was made on the non‐contrast CT images fused with contrast‐enhanced CT images, according to the recommendation of a previous study,^8^ using the Pinnacle treatment planning system (Philips Medical Systems). The gross tumor volume (GTV) was all visible tumors to be treated, and the clinical target volume (CTV) was equal to the GTV in order to minimize the irradiation of noncancerous liver cells. The CTV included liver metastases and their primary lesion when it was active. One or two other metastatic lesions could be included in the CTV whenever considered feasible. The internal target volume (ITV) was defined as the summation of the inspiratory‐ and expiratory‐phase CT images. The planning target volume (PTV) was defined with a 5‐mm margin around the ITV. Delineated OARs were the liver, bilateral kidneys, pancreas, spleen, esophagus, stomach, duodenum, small intestines, colons, spinal cord, heart, and bilateral lungs. The NLV was defined as the whole liver volume minus the GTV and unirradiated lesions.

Subsequent planning and treatments were carried out with the Tomotherapy version 5.0.1 treatment planning station and TomoTherapy HDA system (Accuray, Inc). The TomoHelical mode was exclusively employed. The prescribed dose was 55 Gy in 25 fractions over 5 weeks to cover 50% of the PTV (D_50%_); however, when the gastrointestinal tract was included in the PTV, the dose to those parts was reduced to 50 Gy in 25 fractions. A 2.5‐cm field width was used in the majority of patients. When the irradiation time exceeded 5 minutes, a 5.0‐cm field width was used to shorten the treatment time. A pitch of 0.43 and a normal modulation factor of 2.0 were generally used. The inverse planning was performed with a variable number of iterations, with a range of about 30 to 100 during the optimization process per plan. Our method of helical tomotherapy was previously described in detail.^9^ The fixed jaw mode was used until July 2013. After August 2013, the newly developed dynamic jaw mode was used to reduce the craniocaudal dose spread. The dynamic jaw mode has been shown to be effective in the treatment of various lesion types.^10^, ^11^

### 2.4 Radiation dose constraints for PTV and OARs

PTV constraints were as follows: (a) D_2%_ (near maximum dose) ≤110%; (b) 98% < D_50%_ < 102%; and (c) D_95%_ ≥ 90%. NLV constraints were as follows: (a) V_15Gy_ (% of NLV irradiated ≥15 Gy) ≤55%; (b) V_30Gy_ (% of NLV irradiated ≥30 Gy) ≤30%; and (c) mean dose ≤25 Gy. Constraints for the small intestines (especially the duodenum) were maximum dose <50 Gy. The constraints for other organs were equal to the tolerance dose of normal tissue in 3‐dimensional conformal radiation therapy (3DCRT).^12^ The median PTV was 128 cm^3^ (37‐600 cm^3^). The median NLV was 1175 cm^3^ (720‐2050 cm^3^). The median of mean irradiation dose to NLV was 19.4 Gy (10.4‐25.8 Gy). The median of V_15Gy_ and V_30Gy_ were 45% (20%‐55%) and 23% (10%‐30%), respectively.

### 2.5 DC‐based vaccine therapy and other treatments

DC‐based vaccine therapy was not mandatory, but was allowed according to the wishes of patients. The patients were evaluated for their eligibility for enrollment and the availability of cancer antigens at the immunotherapy clinic (Seren Clinic Nagoya). The methods for the preparation of DC‐based vaccine were based on Kobayashi et al.^13^ DC‐based vaccine was scheduled and administered intradermally every other week while monitoring the condition of the patients. Treatment at recurrence was allowed at the discretion of attending physicians.

### 2.6 Pre‐ and posttreatment evaluation and statistical analysis

Pre‐ and posttreatment evaluation included physical examination, blood tests including tumor markers, and contrast‐enhanced CT and/or MRI. The neutrophil‐to‐lymphocyte ratio (NLR), monocyte‐to‐lymphocyte ratio (MLR), and platelet‐to‐lymphocyte ratio (PLR) in the peripheral blood were calculated before treatment in all patients and within 2 weeks after RT completion whenever possible. Progressive disease of any irradiated tumor according to the Response Evaluation Criteria in Solid Tumors (RECIST) version 1.1 was regarded as local failure. Growth of any lesion including tumors in unirradiated regions was regarded as progression. Toxicity was evaluated according to the Common Terminology Criteria for Adverse Events version 4.0. The criteria for diagnosing radiation‐induced liver disease (RILD) were as follows: (a) ≥3‐fold elevation in serum transaminase or alkaline phosphatase over either the upper normal limit or pre‐RT level; (b) ≥2‐fold serum bilirubin elevation over either the upper normal limit or pre‐RT level; and (c) nonmalignant ascites in the absence of disease progression within 3 months after RT.

OS, PFS, and LC rates were calculated from the start of RT using the Kaplan‐Meier method. Differences in survival curves were analyzed by the log‐rank test. Univariate analysis was performed using Cox's proportional hazards model. All statistical analyses were carried out using an open source software, R Version 3.2.3 (The R Foundation for Statistical Computing). *P* < .05 was considered to indicate a significant difference.

## 3 RESULTS

### 3.1 Overall survival

**Figure 1:**
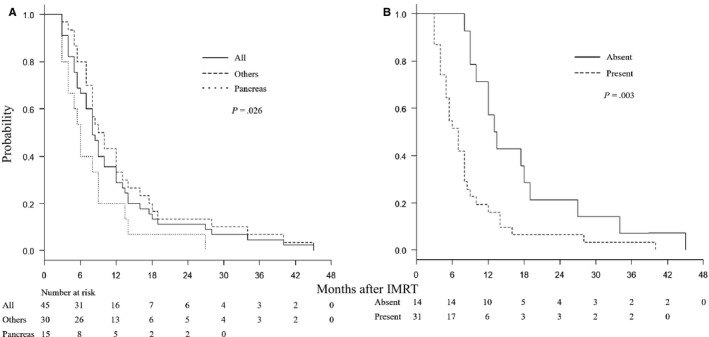
A, OS curves for all patients, those with pancreatic cancer, and those with tumors other than pancreatic cancer. B, OS according to the presence or absence of distant organ metastases outside the liver

**Figure 2:**
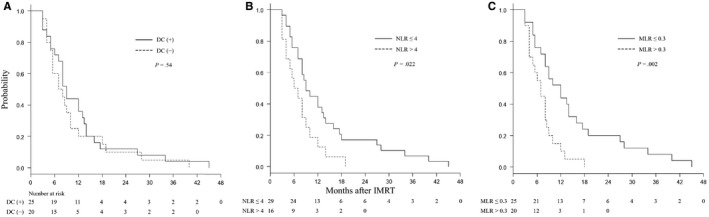
A, OS according to the presence or absence of DC‐based vaccine therapy. B, OS according to pretreatment NLR. C, OS according to pretreatment MLR

Forty‐two of the 45 patients completed the planned treatment. Three patients could not complete the treatment due to worsening of general conditions. The median follow‐up period was 8 months (range, 2‐45 months). All patients died. For all 45 patients, the median survival time (MST) was 8 months, the 6‐month OS rate was 67%, and the 1‐year OS was 29% (Figure 1A). The MST and the 1‐year OS were, respectively, 6 months and 20% for pancreatic cancer patients and 9.5 months and 33% for the other patients. Patients with no lesions outside the liver had better prognosis than those with other lesions (Figure 1B). The MST and the 1‐year OS for patients with DC‐based vaccine therapy were 9 months and 36%, respectively, and those for patients without DC therapy were 7.5 months and 20%, respectively (Figure 2A).

### 3.2 Local control and progression‐free survival

**Figure 3:**
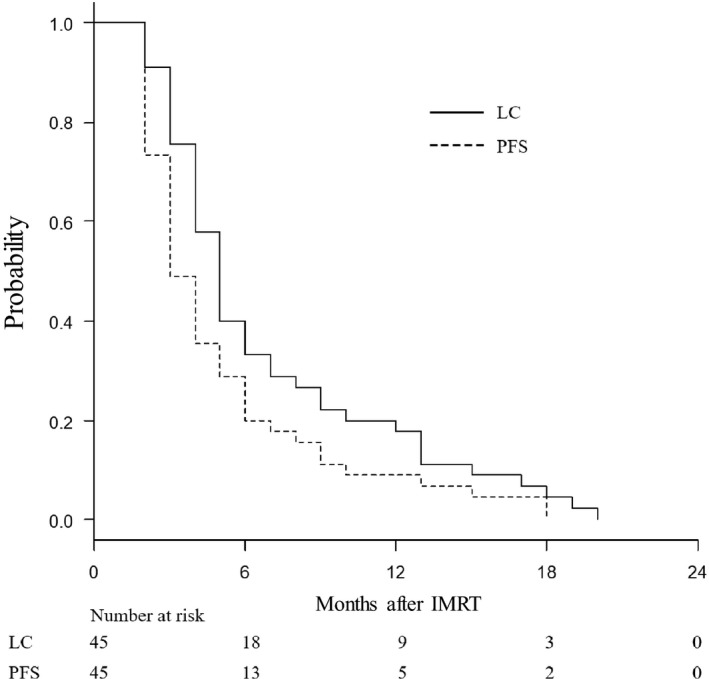
Local control (LC) and progression‐free survival (PFS) curves for all patients

Figure 3 shows LC and PFS curves after tomotherapy. The median LC period was 5 months and the 6‐month control rate of irradiated tumors was 33%. The median PFS time was 3 months and the 6‐month PFS rate was 20%. A marked decrease (≥30%) in tumor marker was observed in 10 (31%) among the 32 evaluable patients with pretreatment tumor marker elevation.

### 3.3 Adverse event

The most common acute toxicity was nausea (71%, Table 2). The most common hematologic toxicity was transient lymphocyte decrease (68%). Two patients with fever developed grade 3 anemia and thrombocytopenia and received blood transfusion. The most common grade 3 toxicity was lymphocytopenia (40%), followed by fatigue (6%). One patient developed grade 3 bile duct stenosis. No patient developed grade 4 or higher toxicity. No apparent RILD was observed. In the group with DC‐based vaccine therapy, no synergy of adverse events was observed.

**Table 2:** Adverse events (n = 45)

Symptom	Grade 1	Grade 2	Grade 3	Total
Nausea	22 (48%)	9 (20%)	1 (2%)	32 (71%)
Vomiting	3 (6%)	1 (2%)	0	4 (8%)
Fatigue	20 (44%)	5 (11%)	3 (6%)	28 (62%)
Fever	0	2 (4%)	0	2 (4%)
Ascites	5 (11%)	3 (6%)	1 (2%)	9 (20%)
Stenosis of bile duct	0	0	1 (2%)	1 (2%)
Cholecystitis	0	1 (2%)	0	1 (2%)
Lymphocyte decrease	4 (8%)	9 (20%)	18 (40%)	31 (68%)
Anemia	3 (6%)	3 (6%)	1 (2%)	7 (15%)
Platelet decrease	8 (17%)	1 (2%)	2 (4%)	11 (24%)
Bilirubin increase	8 (17%)	0	0	8 (17%)
Alanine aminotransferase increase	14 (31%)	2 (4%)	0	16 (35%)
Aspartate aminotransferase increase	19 (42%)	3 (6%)	0	22 (48%)
Alkaline phosphatase increase	15 (33%)	2 (4%)	0	17 (37%)

Note

Adverse events were evaluated according to the Common Terminology Criteria for Adverse Events version 4.0. There was no grade 4 or higher toxicity.

### 3.4 NLR, MLR, and PLR assessments

The median pretreatment NLR, MLR, and PLR were 2.9 (range, 0.8‐8.4), 0.27 (range, 0.05‐0.78), and 142 (range, 43‐874), respectively. The median NLR, MLR, and PLR within 2 weeks posttreatment were 6.5 (range, 2.8‐44.5), 0.81 (range, 0.05‐4.00), and 254 (range, 86‐3200), respectively. Figure 2B,C show OS curves according to the pretreatment NLR and MLR, respectively. A marked transient decrease (≥50%) in lymphocyte counts within 2 weeks after treatment was observed in 24 (68%) of 35 evaluable patients.

### 3.5 Univariate analysis

On univariate survival analysis, primary tumor site (pancreas vs others: *P* = .026), distant organ metastases outside the liver (absent vs present: *P* = .003), pretreatment NLR (≤4.0 vs >4.0: *P* = .022), and pretreatment MLR (≤0.3 vs >0.3: *P* = .002) were significant prognostic factors (Table 3). Regardless of the primary tumor site, DC therapy (with vs without: *P* = .54) was not associated with better prognosis.

**Table 3:** Univariate analysis (n = 45)

Prognostic factor	n	MST	*P* value	HR (95% CI)
		(months)		
Age (y)				
≥65/<65	21/24	8.5/8.0	.26	0.70 (0.38‐1.30)
Sex				
Male/female	25/20	9.0/7.5	.46	1.25 (0.68‐2.30)
ECOG PS				
0‐1/2	29/16	9.0/7.5	.11	1.68 (0.89‐3.15)
Primary tumor site				
Others/Pancreas	30/15	9.5/6.0	.026	2.04 (1.07‐3.87)
Number of liver metastases				
3‐4/ ≥5	7/38	12.0/8.0	.66	1.21 (0.53‐2.74)
Distant organ metastases outside the liver				
Absent/Present	14/31	13.5/7.0	.003	2.71 (1.37‐5.35)
PTV (cm^3^)				
Pancreas (primary tumor site)				
≥150/ <150	6/9	5.0/5.5	.79	0.85 (0.30‐2.47)
Others (primary tumor site)				
≥150/ <150	9/21	7.0/12.0	.056	0.45 (0.20‐1.02)
DC‐based vaccine therapy				
With/Without	25/20	9.0/7.5	.54	1.20 (0.66‐2.18)
Pancreas (primary tumor site)				
With/Without	12/3	8.0/4.0	.069	4.05 (0.90‐18.29)
Others (primary tumor site)				
With/Without	13/17	12.0/8.5	.5	0.91 (0.41‐2.02)
NLR (pretreatment)				
≤4/ >4	29/16	9.0/6.5	.022	2.10 (1.11‐4.00)
MLR (pretreatment)				
≤0.3/ >0.3	25/20	12.0/7.0	.002	2.73 (1.41‐5.27)
PLR (pretreatment)				
<150/ ≥150	22/23	8.5/8.0	.62	1.16 (0.64‐2.11)

Abbreviations: 95% CI, 95% confidence interval; DC, dendritic cell; ECOG, Eastern Cooperative Oncology Group; HR, hazard ratio; MLR, monocyte‐to‐lymphocyte ratio; MST, median survival time; NLR, neutrophil‐to‐lymphocyte ratio; PLR, platelet‐to‐lymphocyte ratio; PS, performance status; PTV, planning target volume.

### 3.6 Retreatment for recurrence

Six received the second tomotherapy, one received the third tomotherapy, and one received the fourth tomotherapy for new liver metastases outside of the irradiated volume. None of the patients undergoing repeat tomotherapy developed RILD. Systemic chemotherapy was attempted in 12 patients due to recurrence.

## 4 DISCUSSION

The role of RT in the treatment of liver metastases has been limited, whereas recently, SBRT and particle therapy have been applied to oligometastatic liver tumors and favorable outcome has been reported.^1^, ^2^ For multiple liver metastases not indicated for SBRT or particle therapy, whole liver irradiation is currently not being applied unlike the brain because of the low tolerance to radiation of the noncancerous liver cells. Whole liver irradiation was previously attempted as a prophylaxis of liver metastases with the dose around 20 Gy, resulting in decreased occurrence of liver metastases and elongated survival.^14^ Palliative 3DCRT against the whole liver has also been used for multiple liver metastases in the past in Japan. Generally, the liver is sensitive to radiation and up to 30‐35 Gy against the whole liver is considered to be the safe limit.^15^ These doses are not high enough to obtain long‐term control of liver metastases. On the other hand, recent developments in radiotherapy technology have enabled treatment of multiple liver metastases with IMRT. Helical tomotherapy may be one of the best modalities for this purpose. The present study demonstrates the feasibility of treating 3‐10 tumors with a dose of 55 Gy in 25 fractions with no RILD. Even repeat treatment of the liver for new multiple metastases was feasible and tolerable in eight patients. Therefore, if the efficacy could be demonstrated, this method has the potential to become a new treatment for multiple liver metastases.

The MST of 8 months and the 1‐year survival of 29% seem to be favorable for the patients with chemo‐refractory liver metastases, since the expected survival time at the start of tomotherapy was estimated to be several months if untreated. In most previous studies on systemic chemotherapy for metastatic pancreatic and biliary tract cancer, the MST was less than 1 year,^16^, ^17^, ^18^, ^19^ although the MST was longer than 2 years when liver metastases from colorectal cancer became resectable after chemotherapy.^20^ In the present study, the median duration of systemic chemotherapy was 10 months (7 months for pancreatic cancer metastases) and the MST after tomotherapy was 8 months (6 months for pancreatic cancer metastases), so OS times after chemotherapy in our patients compare favorably with those after chemotherapy alone in the previous reports.^16^, ^17^, ^18^, ^19^ The median LC period for irradiated tumors and PFS time were 5 and 3 months, respectively; since all the tumors were progressing at the start of tomotherapy, these results would also indicate the modest efficacy of the treatment. The dose of 55 Gy in 25 fractions may be insufficient to obtain long‐term control, especially for metastases from colorectal cancer, but prolongation of OS seemed to have been obtained. Preirradiation chemotherapy may increase the efficacy of RT,^21^ but this might not apply to our cases because all the tumors had become chemo‐refractory. Regarding pancreatic cancer, the MST was 4.5 months and the 1‐year survival was 14.1% in a Japanese phase II study of S‐1 in gemcitabine‐refractory metastatic pancreatic cancer.^22^ Considering this extremely poor outcome, an MST of 6 months and the 1‐year survival of 20.0% for gemcitabine‐refractory pancreatic cancer are a favorable outcome.

In attempting such a treatment, toxicities were a concern, since considerable volumes of noncancerous liver cells were irradiated. Remarkably, liver function was preserved in all cases. The most common acute adverse events were grade 1 or 2 nausea and vomiting. The gastrointestinal tract is considered to be a main reservoir of serotonin and radiation induces damage to the mucous membrane, leading to the release of the serotonin.^23^, ^24^ Serotonin activates 5‐hydroxytryptamine‐3 (5‐HT3) receptors, mediating nausea and vomiting.^25^ In current antiemetic guidelines, RT to the upper abdomen is classified in the moderate emetogenic risk group.^26^, ^27^ The guidelines recommend the administration of a 5‐HT3 receptor antagonist to prevent nausea and vomiting. In the present study, a 5‐HT3 receptor antagonist was only administered to two of 32 patients developing nausea and vomiting. Hereafter, the early administration of this kind of drug should be considered after the start of treatment.

Another concern was radioresistance of the tumors acquired from preceding chemotherapy. Cross‐resistance to radiation in tumors treated by chemotherapy (especially DNA‐damaging agents) has been reported.^28^, ^29^, ^30^ Most of the chemotherapy regimens used before tomotherapy in this study included DNA‐attacking agents, so it was a concern that radiation might not provide sufficient effects. Previous studies indicated that acquisition of cross‐resistance depended on the cell line and was related to an increase in the glutathione level.^29^, ^30^ Nevertheless, we obtained a partial or complete response according to RECIST in 64% of the 42 evaluable tumors (data not shown in RESULTS). Therefore, it appears worthwhile to attempt RT for chemo‐refractory tumors. In addition, combining IMRT with chemotherapy in the first‐line treatment of multiple liver metastases may be a topic of future investigation. In this study, the presence of distant metastases outside the liver was a negative prognostic factor. Intensive treatment could potentially improve the prognosis in patients with metastases confined to the liver.

In this study, the contribution of DC therapy to OS was unclear. In a review of DC therapy, various reasons for the limitations of the treatment's efficacy were suggested, including inhibition of immune responses by regulatory T cells (Treg) and increase of the Treg number by transforming growth factor‐β (TGF‐β).^31^ The elevated expression of TGF‐β1 was reported after whole or partial liver irradiation in in vivo studies.^32^, ^33^ Therefore, irradiation to the normal liver using the TomoHelical mode might lead to increases in TGF‐β1 and Treg number, decreasing the efficacy of DC‐based vaccine therapy. It was reported that the pretreatment NLR, MLR, and PLR were associated with the prognosis of patients with malignant solid tumors.^34^, ^35^, ^36^ In a systematic review and meta‐analysis, NLR greater than 4 was associated with the poor prognosis,^34^ and this was also the case in our study. In immunotherapy, it was also reported that baseline and early changes in NLR, MLR, and PLR were strongly associated with clinical outcomes in patients with advanced cancer.^37^ Lymphocytes in the blood, vertebral bone marrow, spleen, and small intestines are radiosensitive. Hepatic irradiation did not affect intrahepatic lymphocytes in an in vivo study.^38^ Accordingly, the irradiated regions outside the liver could lead to decrease in lymphocytes, resulting in the increase of NLR and MLR in the early stages of posttreatment.

Based on these new findings and outcomes of the present study, further decrease of low‐dose regions outside the liver seems more important in order not to cause a decrease in the lymphocyte count. As a disadvantage of the TomoHelical mode, the regions receiving low‐dose radiation are generally broad compared with those of 3DCRT. In recent years, the use of the TomoDirect mode has been spreading and the technical efficacies are being ascertained.^39^, ^40^ Depending on the location of liver metastases, using the TomoDirect mode could be suitable to reduce low‐dose regions outside the liver and could prevent a decrease in the host immunity. A planning study evaluating the TomoDirect mode for liver metastases is ongoing.

In conclusion, this study suggested that helical tomotherapy for chemo‐refractory multiple liver metastases was a feasible and potentially effective treatment with acceptable adverse events. Additional systematic studies are required to evaluate the optimal combination methods of DC‐based vaccine therapy and RT for optimal survival benefits.

## CONFLICT OF INTEREST

The authors declare no conflict of interest.

## Data Availability

The data that support the findings of this study are available on request from the corresponding author. The data are not publicly available due to restrictions e.g. their containing information that could compromise the privacy of research participants.
